# Developing neuroscience-based treatments for alcohol addiction: A matter of choice?

**DOI:** 10.1038/s41398-019-0591-6

**Published:** 2019-10-08

**Authors:** Markus Heilig, Eric Augier, Simone Pfarr, Wolfgang H. Sommer

**Affiliations:** 1 Center for Social and Affective Neuroscience, Department of Clinical and Experimental Medicine, Linköping University, S-581 83 Linköping, Sweden; 2 Institute of Psychopharmacology, Central Institute of Mental Health (CIMH), J 5, 68159 Mannheim, Germany; 3 Department of Addiction Medicine, Central Institute of Mental Health (CIMH), J 5, 68159 Mannheim, Germany

**Keywords:** Addiction, Molecular neuroscience

## Abstract

Excessive alcohol use is the cause of an ongoing public health crisis, and accounts for ~5% of global disease burden. A minority of people with recreational alcohol use develop alcohol addiction (hereafter equated with “alcohol dependence” or simply “alcoholism”), a condition characterized by a systematically biased choice preference for alcohol at the expense of healthy rewards, and continued use despite adverse consequences (“compulsivity”). Alcoholism is arguably the most pressing area of unmet medical needs in psychiatry, with only a small fraction of patients receiving effective, evidence-based treatments. Medications currently approved for the treatment of alcoholism have small effect sizes, and their clinical uptake is negligible. No mechanistically new medications have been approved since 2004, and promising preclinical results have failed to translate into novel treatments. This has contributed to a reemerging debate whether and to what extent alcohol addiction represents a medical condition, or reflects maladaptive choices without an underlying brain pathology. Here, we review this landscape, and discuss the challenges, lessons learned, and opportunities to retool drug development in this important therapeutic area.

## Alcohol addiction: an area of large unmet medical needs

Excessive alcohol use accounts for about 5% of global disease burden and close to 6% of all deaths^1,2^. By many measures, the harm from alcohol exceeds that from illicit drugs^3^. A population of alcohol-addicted people with very heavy drinking, defined as >100 or 60 g/day for males or females, respectively, are disproportionately affected^4^. This population, ~0.8% of people aged 15–65 years in a group of European countries, accounts for about half of all liver cirrhosis cases, and has a life expectancy that is dramatically shortened, by 25—31 years.

Only about 25% of people with alcoholism ever receive treatment; for those who do, the lag from filling diagnostic criteria to receiving treatment is about a decade^5^. This treatment gap is in large part caused by a lack of effective treatments with good patient acceptance. Behavioral treatments with support for efficacy exist, but their effect sizes are modest^6^. Pharmacotherapies for alcoholism are few, have limited efficacy and patient acceptance, and their uptake in clinical practice is minimal^7^. It is frequently claimed that alcoholism medications are not developed because their commercial potential would be small, but data suggest otherwise. The market in alcoholism treatment provision has been estimated to ~$35 billion/year in the US alone^8^. However, treatment for the most part takes place outside the medical system. A lack of medications with robust effect sizes and good patient acceptance is a major reason for this situation, but other factors, such as insufficient physician training in addiction medicine, also contribute.

## Current options

Medications with three mechanisms are currently approved for the treatment of alcohol addiction by the European Medicines Agency (EMA) or the Food and Drug Administration (FDA). The oldest, the aldehyde dehydrogenase inhibitor disulfiram, remains most widely used. Its mechanism of action is peripheral, and cannot alleviate craving or other subjective states associated with alcoholism. Its use is associated with low compliance, and does not have support for significant efficacy unless administered under supervision^9^. Disulfiram continues to have a place in clinical practice when sobriety needs to be ensured for some time, for instance to diagnose psychiatric co-morbidity. It is otherwise of limited interest for the present discussion.

Proof-of-principle for a neuropharmacological intervention in alcohol addiction was first provided by the mu-preferring opioid antagonist naltrexone^10–12^. Opioid peptides with activity at mu-receptors are released by alcohol intake, and contribute to alcohol reward^13–16^. Naltrexone is thought to interfere with this cascade. The overall effect size of naltrexone is modest^7^, but this represents an average of a heterogeneous response, that varies strongly as a function of individual patient characteristics. Among these characteristics, predictors of clinical response include a family history of alcohol problems, early onset of problem drinking, being male, experiencing strong alcohol reward-related memories or cravings, and complying with treatment^17,18^. The role of compliance can be viewed in light of extensive empirical data in support of the notion that opioid transmission plays a key role for the “liking” of natural rewards^19^. Based on these findings, it can be hypothesized that naltrexone has a potential to attenuate healthy rewards, and that this limits the incentive to seek and comply with this treatment. A depot formulation of naltrexone was developed to improve compliance, but high cost limits its use. Another opioid antagonist approved for alcoholism treatment in Europe, nalmefene^20^, shares its main mechanism of action with naltrexone, making major differences in clinical profile unlikely.

The homo-taurine analog acamprosate is approved for the treatment of alcoholism, and its efficacy is supported by meta-analysis. The effect size is however small, and was not reproduced in the largest trial^7,21^. Both in animal models and in patients, acamprosate influences glutamatergic mechanisms^22,23^. Animal data suggest that severely dependent individuals, in whom glutamatergic dysregulation is most pronounced, should be most likely to respond to acamprosate^24^. However, the exact molecular mechanism through which acamprosate exerts its effect remains unknown, and it has been suggested that it simply functions as a carrier of calcium-ions into the CNS^25^. This mechanism could help explain its low potency; the recommended daily dose is 2 g. The combination of modest efficacy, a short half-life that necessitates three times daily administration, and a high frequency of gastrointestinal side effects has led to a very limited clinical uptake of acamprosate.

Meta-analytic support for efficacy is also present for some medications that lack approval for the treatment of alcohol addiction, but are approved for other indications. Among these, perhaps the strongest support is available for the anti-epileptic topiramate^26–28^. Topiramate has complex molecular mechanisms of action, but because its efficacy appears to be moderated by a polymorphism at the locus encoding the kainate receptor subunit GRIK1^29^, its actions are likely to be mediated through glutamatergic mechanisms. The anti-emetic 5HT3 antagonist ondansetron has support for efficacy in some patient populations^30–32^. There are additional approved medications that have some support for their efficacy in alcohol addiction, suggesting that they could be considered for repurposing. These include the nicotinic partial agonist varenicline^33,34^ that has marketing approval for smoking cessation, and the GABA-B agonist baclofen^35–42^, which has long been used for spasticity.

Several medications approved for the treatment of alcohol addiction, and a few more with data supporting their off-label use might not seem so bad. The realities on the ground unfortunately paint a different picture. The medications reviewed in the section above are prescribed to a vanishingly small fraction of alcohol-addicted patients. Even within the US Veterans Health Administration, where prevalence of alcohol use disorders is high, and where systematic strategies for their identification are in place, only about 3% of patients diagnosed with alcoholism receive any prescription medication, while corresponding numbers have been estimated to 0.07% overall and 5.8% of those seeking specialty treatment elsewhere^43,44^. A quarter century of considerable public investment in research funding, and of academic efforts to bring forward pharmacotherapies for alcohol addiction have had little if any impact on the real world of patients. This is arguably one of the key facts behind a growing backlash against the concept of addiction as a brain disease^45^.

## A translational crisis

The disease burden from alcohol addiction, and the inability of existing medications to significantly improve outcomes in this condition provide a strong incentive for research to identify new pharmacotherapeutic mechanisms. With this as a rationale, the past two decades have seen an exponential growth in neuroscience studies on alcohol addiction. The expansion has in large part been driven by the US National Institute on Alcohol Abuse and Alcoholism (NIAAA), which made it a priority to promote neuroscience aimed at ultimately addressing treatment needs^46,47^. The task at hand is as important as it is challenging. Alcohol acts on a multitude of brain targets, including multiple ionotropic receptors for GABA and glutamate, as well as metabotropic receptors for GABA, glutamate, dopamine, and endogenous opioids^48^. It is presently unclear which among these actions that underlie the initiation, progression, and maintenance of alcohol addiction. Identifying mechanisms that can be targeted by novel alcoholism treatments therefore poses a major challenge.

The quantitative growth in neuroscience of alcohol addiction has combined with major technical and methodological advances to give cause for optimism. Across the field of addiction, increasingly sophisticated tools have allowed researchers to identify putative neural substrates of drug reward, protracted withdrawal and relapse using animal models^49–55^. Optogenetic and chemogenetic methods, combined with sophisticated gene-targeting tools and in vivo visualization methods have opened up new avenues of research that can precisely define and control neural circuits that underlie these behaviors^56–59^. This research typically utilizes models in which laboratory animals self-administer drugs^60^, widely regarded as valid because most drugs abused by humans are also self-administered by rodents and monkeys^61^.

Yet for all these advances, improved alcoholism treatments have not emerged. In fact, some of the most promising therapeutic mechanisms identified by basic research have failed in clinical development^62–64^. Overall, neuroscience has simply had very little impact on clinical alcoholism treatment^65,66^. This situation is representative of a broader translational crisis in psychiatric neuroscience. Because translational failures in this area have been the rule rather than the exception, pharmaceutical industry has largely retreated from efforts to develop novel psychiatric medications^67^. As a result, the utility of animal models in research on psychiatric disorders, including addiction, is also being questioned^68^.

It should also be noted that a deep understanding of a drug’s mechanism of action is in no way a *condition sine qua non* to therapeutic efficacy. After all, the site of action of substances, such as cocaine has been known for decades and yet there is no better advancement in therapeutics for cocaine addiction. Also, alcohol’s known actions on the dopamine system have been targeted many times, with negative or even detrimental outcomes in all these trials (reviewed in ref. ^14^). It has become clear that, without the ability to determine the specific state of an individual’s dopamine system and its responsivity, direct pharmacological interference with DA receptors and transporters is unlikely to succeed as a viable treatment approach.

The failure so far to achieve more than at best marginal gains in alcoholism treatment through pharmacotherapy strongly suggest that business as usual in alcoholism research should not be considered an acceptable option. Non-medical strategies, such as addressing societal factors that promote addiction^65^ and implementing policies that limit alcohol consumption^69^ should of course be pursued to reduce the disease burden from alcohol use, but do not replace effective medical treatments for people who nevertheless develop alcohol addiction, in particular the population of people with dependence and very heavy drinking^4^.

## But is there a disease to treat?

The hopes that neuroscience would bring novel treatments to patients with addictive disorders is intimately related to the conceptualization of addiction as a chronic relapsing brain disease^70^. In recent years, this conceptualization has encountered increasing criticism^45^. One line of argument is that high spontaneous remission rates are inconsistent with a disease-oriented view of addiction. According to a re-analysis of US population data^71^, about half of individuals with a diagnosis of alcohol dependence remitted within 20 years^72^. This was taken to provide support for a need to re-conceptualize addiction as a “disorder of choice”. According to that view, rather than being compelled by a disease, drug seeking and taking is best accounted for by models in which people with addictions make choices that are based on cost-benefit trade-offs, and that are in principle no different from choices made by people without addictive disorders.

Efficacy of treatment approaches such as contingency management, which provides systematic incentives for abstinence^73^, certainly supports the notion that behavioral choices in patients with addictions including AUD^74^ remain sensitive to reward contingencies. It is unclear, however, to what extent this invalidates a “brain disease model of addiction”. The critical question would seem to be whether addictive behaviors—for the most part—result from healthy brains responding normally to externally determined contingencies, or rather from a pathology of brain circuits that promotes suboptimal choices even when reward contingencies are within a normal range.

Conceptualizing addictive behaviors as choices that are sensitive to incentives emphasizes the importance of cognitive function and decision making over a narrow focus on classical reward circuitry. This perspective is neither new, nor foreign to mainstream neuroscience of addiction^75–79^.

While impaired executive function may have many causes including, drug and non-drug reinforcer specific mechanisms of actions, it is plausible to speculate that some of these mechanisms converge on common neurobiological substrate that can be identified and targeted. For example, alcohol causes long-lasting alterations to glutamatergic neurons in the mPFC including loss of metabotropic glutamate receptor 2 (mGluR2) that can be causally linked to the increased reinforcing properties of the drug^80^, but are likely to impact on executive function as well. Common mechanisms underlying pleitropic phenomena, such as cognitive function and decision making are also suggested by the key role these systems play in contemporary treatment approaches across all addictions, such as cognitive-behavioral coping skills therapy^6^, interoceptive training to improve attention and emotional regulation^81,82^, or strengthening executive control^79^.

It is clear that these approaches can be clinically efficacious, even though their effect sizes are modest. What is not clear is that an important role for decision-making systems in addictive behaviors would provide an argument against a disease view of addiction. Economic theory recognized more than a quarter century ago that rational choice is insufficient to explain human choices in general, and that systematic biases consistently result in suboptimal decisions in most healthy people^83^. The question is whether, in people with addiction, biases in decision making are quantitatively or qualitatively different from those in people without this condition. This does indeed seem to be the case. For instance, excessively discounting temporally distant rewards is a characteristic of patients with various addictive disorders, reflects the quantity of drugs used, and predicts treatment outcomes^84^. Notably, discounting rates can be modified by training or pharmacology, potentially offering opportunities for development of novel therapeutic interventions. Consequently, delay discounting of rewards has been suggested as a biomarker of addiction and its treatment^85,86^.

Nevertheless the mechanistic understanding in this area is rather limited, and again one approach does not fit all. The domains for which biomarkers such as delay discounting are proposed for, need to be further deconstructed, mapped to clinical symptoms, and evaluated for predictive validity. For example, in rodents amphetamine improves performance in delay discounting tests but worsens premature responding in a stop signal task (a common measure of motor impulsiviry), while atomoxetine has the opposite effects^87^. Yet, despite these very different profiles, the two drugs both have documented clinical efficacy to reduce impulsivity in patients with ADHD. Another promising drug to improve impulsivity measures is modafinil, a wakefulness-promoting drug approved for narcolepsy, that has shown some beneficial effects in cocaine and methamphetamine-dependent patients^88–90^. Modafinilin has been tried in alcoholics. Notably, clinical drinking outcomes were dependent on baseline patient characteristics: Patients with initially poor inhibitory control showed improvement in abstinence and drinking rates, but those with lower baseline impulsivity scores worsened in response to the same treatment^91^.

Unless we resort to a mind–body dualism, systematic biases in choice behaviors that distinguish people with addictive disorders from healthy subjects must have a basis in a neural pathology that is pre-existing, results from drug exposure, or—most likely—both. Several features of clinical addiction do provide a strong argument against the proposition that continued substance use despite adverse consequences becomes a rigid, stimulus-driven behavior that merits the label “compulsive”^92^. Examples of such features are high rates of spontaneous remission, preserved sensitivity to contingencies, and efficacy of treatments that strengthen cognitive control. These features do not, however, necessarily invalidate a view of addiction as a brain disease. A balanced view would seem to suggest that to some extent, value-based decision making is preserved in patients with addiction, but that systematic biases different from those found in healthy people are present in this population when it comes to outcome valuation and action selection. This indicates a pathology of brain circuits that are used to generate, and operate on, models of the world.

We maintain that in order to prove its value, a “brain disease model of addiction” will have to bring forward effective treatments. To do so, it will have to identify neural and molecular mechanisms that account for systematic biases in alcohol-related choice behaviors. This in turn will require a mechanistic understanding of how drug-associated memories are encoded, retrieved and altered, and how they guide action selection. This mechanistic understanding will then need to be utilized to develop treatments that can help patients shift decision-making biases back toward more adaptive choices.

## Substrates of decision making in alcohol addiction

The prefrontal cortex plays a major role in decision-making processes and cognitive functions that exert top-down control over motivational and emotional drives. Accordingly, the past decade has seen an increased interest in this structure and its role in addiction in general^77,81,93^. This has coincided with seminal work which established the structural and functional diversity of rodent prefrontal areas, their relation to the primate brain, and their role in attentional and emotional processing^94–96^. For the discussion here, we will focus on the medial prefrontal cortex (mPFC), in primates mostly composed of the anterior cingulate cortex (ACC) and its major divisions: anterior midcingulate (aMCC), pregenual (pACC) and subgenual (sACC), also referred to as Broddman areas 24, 32, and 25, respectively. The corresponding rodent areas are the cingulate (Cg), prelimbic (PL), and infralimbic (IL) cortices, respectively^97^. In rodents, the IL seems especially vulnerable to the neurotoxic effects of chronic alcohol intoxication, as shown by reduced expression of glutamatergic marker genes and a reduction in the total volume of neurons^80,98^.

Anatomical localization, however, turns out to be insufficient to characterize the functional involvement of these structures. As a result of methodological advances, the concept of neuronal ensembles postulated by Hebb more than half a century ago^99^ has returned in full force^59^. It is rapidly becoming clear that this concept is critical for understanding valuation and decision-making processes that ultimately result in drug or alcohol use. Rather than reflecting activity of entire brain structures, such as the mPFC or the amygdala, these processes seem to reflect the activity of small neuronal populations that are sparsely distributed within the respective structure^100–102^. Functional ensembles are characterized by coordinated spatiotemporal activity and reliable re-activation during specific behavioral tasks. Neuronal ensembles are dynamic, meaning that they are formed and updated throughout the learning of a behavior^103^. Selection of cells that become included in an ensemble also depends on the activity pattern of afferent inputs, supporting the notion that ensembles located in different brain areas interact^59,104^. Taken together, an individual neuron can be part of multiple ensembles that are involved in different behavioral responses. At the same time, individual ensembles can be distributed across multiple brain structures, while individual brain structures can harbor neuronal ensembles that encode different behaviors.

Detailed spatial maps of temporally coherent neural activity can be obtained by histological methods. For this purpose, expression of the immediate-early gene cFos induced in strongly activated neurons can easily be detected^105^ and linked to behavioral output^59,104^. Early studies using antisense oligonucleotide approaches demonstrated functional consequences of blocking cFos expression on behavioral, neurochemical, or electrophysiological responses^106–108^. More recently, the use of transgenic animals has allowed investigators to selectively label and delete ensembles of neurons that are active during a specific behavior^59,104^. These studies have used a Fos-LacZ-rat line that expresses the bacterial enzyme beta-galactosidase under the control of the cFos promoter. In neurons that are strongly activated, induction of Fos-expression in this line is accompanied by expression of the enzyme beta-galactosidase. If the prodrug Daun02 is subsequently injected into a brain area, beta-galactosidase converts the prodrug into the neurotoxin daunorubicin, resulting in apoptotic cell-death of the previously activated cells, but not other neurons^101,109,110^.

Activity-dependent neuronal tagging and deletion using Daun02 has allowed identifications of neuronal ensembles involved in cocaine, heroin, alcohol, and food seeking^58,101,111–113^. The functional ensemble that is activated within the infralimbic mPFC upon recall of an alcohol memory comprises about 10–15% of IL neurons^101^. After Daun02 inactivation of this ensemble, rats dramatically increased their alcohol seeking, in what can be viewed as a loss of control over drug seeking. This was not seen after non-selective inactivation of the IL, indicating that the functional output of a brain region relies on specific ensembles, rather than the general activity of the region. Also, despite similar alcohol-cue-induced cFos activation in the neighboring prelimbic cortex, Daun02 administration in this area had no behavioral consequences on alcohol seeking. Finally, these experiments provided evidence for the prediction that diverse functional ensembles can co-exist within the same brain region. Specifically, both exposure to alcohol-associated cues and stress induces infralimbic cFos expression. However, Daun02 inactivation after cue-exposure only affected the cue-induced behavioral response, leaving the response to stress unaffected. In general agreement with these findings, others have found that distinct ensembles within the infralimbic cortex can concurrently be activated by different environmental cues, and control opposing behavioral outputs, i.e. approach and avoidance^112–114^.

Initial investigations into ensemble formation and organization under choice conditions, i.e. animals could choose between different rewards, revealed highly similar and largely overlapping cFos activation patterns. Specifically, we employed a two-reward operant-conditioning procedure for alcohol and saccharine seeking in combination with a newly developed double fluorescence in situ hybridization method that allowed the detection of two independently elicited cFos responses. After two successive brief cue exposure sessions for each reward, we detected about 50% overlap between the corresponding ensembles^100^. Although this approach so far does not allow to study real-time decision making, the initial results suggest highly overlapping representations of drug and natural reward-related memory traces, at least in the infralimbic cortex.

This finding is not unexpected. In a previous study, functional imaging in rats showed similar activation maps in the mesocorticolimbic system after voluntary consumption of a sweet saccharin or of 10% alcohol solution^115^. Similarly, a meta-analysis of 176 human cue-reactivity studies revealed overlapping neuronal activations to be associated with craving for natural and drug rewards^116^. Further meta-analyses implicate particularly the mPFC in alcohol cue-reactivity and relapse risk^117,118^. In fact, the mPFC has recently been suggested as common ‘hot spot’ of cue-reactivity, making its activation a potential transdiagnostic endophenotype of addiction, and point to the PFC as a fruitful neuromodulation target^119^. It is evident that target selection for brain stimulation treatment development requires deeper mechanistic understanding. An important question to resolve is how aberrant or pathologically heightened drug cue reactivity can be disentangled from neural responses to natural and healthy reward cues in this area.

Ensembles encoding aspects of alcohol memories that are important for reward learning, valuation, and action selection are not confined to the mPFC. Based on the multitude of neuroimaging studies, (recently reviewed in ref. ^120^), contributions to the behavioral outcome of decision making are expected to come from various prefrontal areas, such as ACC, OFC, and insula, as well as from subcortical reward-related areas, e.g. amygdala or striatum. Neuronal ensembles in most of these structures that are causally related to drug seeking, choosing, and taking have not yet been identified. However, using the Daun02 method, a neuronal ensemble in the central nucleus of the amygdala (CeA) was recently identified that is critical for alcohol self-administration during withdrawal from alcohol dependence^102^. Furthermore, a recent study used a variant of the Daun02 method, in which LacZ expression in a transgenic mouse line is driven by the promoter of the inflammatory mediator nuclear factor (NF)-κB. In the course of classical reward learning, acquisition of conditioned place preference for alcohol, cells activated in the nucleus accumbens by a rewarding dose of alcohol were deleted. This prevented acquisition of the reward memory, as showed by failure to develop place preference^121^.

Circuit-specific manipulations using chemogenetic or optogenetic approaches will allow further mechanistic insights that also might guide neuromodulation-based therapies for addictive disorders^122,123^. For instance, a recent study in rats indicated that a history of alcohol dependence alters the functional connectivity of the anterior insula^124^. Chemogenetically silencing this structure using a DREADD (*d*esigner *r*eceptor *e*xclusively *a*ctivated by *d*esigner *d*rug) changed alcohol’s interoceptive properties and approach behavior to the drug^125^. We believe that circuits-based manipulations need to be combined with functional neuroimaging both in animals and humans to better understand the effects of local interferences on large-scale brain network properties.

## Choice and individual vulnerability

Understanding the pathology of brain circuits that promotes choice of alcohol over healthy rewards is likely to be key for understanding the mechanisms of alcohol addiction, and targeting them with therapeutics. However, only a minority of people transition from recreational to addictive alcohol use, a pattern that is similar to that of other addictions^126,127^. In contrast, in animal self-administration and relapse models, nearly all rats acquire the respective behaviors, and individual variation is treated as random experimental error. If pathophysiological mechanisms of alcohol addiction are unique to a vulnerable minority, than these commonly used research strategies may be inherently limited in their ability to identify clinically relevant treatment targets.

Attempting to address these limitations, we have started to examine individual variation in choice behavior^128^. When genetically heterogeneous Wistar rats were first trained on oral alcohol self-administration and then assessed in a mutually exclusive discrete trial procedure, the vast majority of rats showed a choice preference for a non-drug reward, an intensely sweet saccharin solution. However, a stable minority or ~15% of the population chose alcohol over the non-drug reward. These rats showed other interesting behavioral characteristic. For instance, their motivation to obtain alcohol was elevated when assessed by break-points on progressive ratio schedule self-administration. Furthermore, and similar to what is seen in clinical populations, their self-administration was insensitive to aversive consequence, such as adulteration of the alcohol solution with quinine or contingent administration of foot shock.

In search of molecular targets for novel alcoholism medications, we compared transcriptome profiles between the minority of rats that chose alcohol over the natural reward, and the majority that did not. We analyzed differential gene expression in multiple brain regions commonly implicated in alcohol addiction, including the prelimbic and infralimbic mPFC, as well as subcortical structures, such as the nucleus accumbens and the amygdala. Using this strategy, we found little evidence for differential gene expression in most brain structures analyzed. The exception was the amygdala, where we discovered differential expression of multiple genes related to GABA-signaling as a characteristic of rats with a choice preference for alcohol^128^. Interestingly, activation of a neuronal ensemble within the CeA has previously been reported to be associated with escalated alcohol self-administration in alcohol-dependent rats. Daun02 inactivation of this ensemble during abstinence resulted in a long-lasting reduction of alcohol drinking^102^.

Our findings of altered expression in a range of GABA-ergic genes associated with alcohol choice and aversion-resistant alcohol use converged with prior electrophysiological studies in rats^129^, and meta-analytically confirmed human genetic findings^130^. The most prominent among the gene expression findings was a low amygdala expression of the GABA-transporter GAT-3 in rats with alcohol choice preference. A translational validity of this finding was suggested by the fact that GAT-3 expression was also low in post-mortem central amygdala from people with alcoholism. In rats, low GAT-3 expression is causally related to alcohol choice behavior, because a knockdown of GAT-3 was able to convert rats that originally did not choose alcohol over the natural reward to doing so.

It is presently unknown whether the changes in gene expression discovered in our choice experiments are the result of DNA sequence variation, epigenetic reprogramming or both. Multiple mechanisms could converge to result in pathological alcohol choice behavior. Pre-existing vulnerability factors can promote alcohol choice, but prolonged heavy drinking contributes to the development of alcohol addiction per se. On a group level, prolonged exposure of the brain to cycles of alcohol intoxication and withdrawal induces persistent neuroadaptations that in turn promote alcohol seeking, taking, and relapse^131–133^. This is in part mediated through epigenetic reprogramming of the transcriptome in key brain regions^76,134–138^. A key question ahead is whether a history of physical alcohol dependence increases the proportion of rats that choose alcohol over an alternative reward, and if so, whether dependence-induced neuroadaptations exert their influence on alcohol choice through the same GABAergic mechanism in the amygdala as those that mediate pre-existing vulnerability.

Integrating the role of mPFC, nucleus accumbens and central amygdala in alcohol choice will require extensive additional research. It is however clear that the amygdala, traditionally associated with emotion and motivation, is also heavily involved in representing value^139^.

## Implications for treatment development

If alcohol memories are encoded by distinct neuronal ensembles, then selectively targeting these ensembles might hold therapeutic promise. Deleting neurons activated in conjunction with recall of alcohol memories, using methods patterned on the Daun02 approach, is unlikely to be realistic or even desirable in a clinical context, but manipulating these ensembles in less drastic ways may be possible. Treatments that aim to extinguish the association between conditioned stimuli and maladaptive responses have long been in clinical use, both outside the addiction field, such as prolonged exposure therapy (PE) for post-trauamatic stress disorder (PTSD)^140^ and specifically for the treatment of alcohol addiction^141,142^. Unfortunately, their efficacy is limited, their effects are to a large extent context-dependent, and the maladaptive responses frequently show renewal over time. This is thought to reflect the fact that extinction is a learning process per se, and creates a new memory trace rather than eliminating the original one^112,143^. To overcome the limitations of simple extinction training, attempts have been described to modify or erase the original memory traces. These approaches are based on the notion that when a memory is retrieved, it becomes labile for several hours and requires reconsolidation before once again becoming stable, thus providing a window for interventions to degrade the memory trace^144–146^. This is a concept that is as appealing as it is controversial^147^.

Based on this framework, it has been suggested that pharmacological interventions might enhance memory reconsolidation—erasure procedures. For instance, reconsolidation of alcohol-associated memories was weakened in rats by the non-competitive N-methyl-D-aspartate (NMDA) receptor antagonist MK-801^148^. Using an established retrieval-extinction protocol, this group then went on to show that the partial NMDA receptor agonist D-cycloserine potentiated “memory erasure”, resulting in a decreased reactivity of ventral striatum to alcohol cues, and a decrease of relapse-like behavior^142^. The beta-adrenergic antagonist propranolol has also been proposed to influence memory reconsolidation^149^. Propranolol has been reported to potentiate the effects of a retrieval-extinction procedure on measures of preference for nicotine-associated conditioned cues and nicotine craving in people, and on relapse to nicotine-conditioned place preference and operant nicotine seeking in rats^150^. In rats, the retrieval manipulation also activated distinct neuronal ensembles in the basolateral amygdala that control nicotine preference and seeking^151^. However, studies with propranolol in alcohol-addicted patients are so far lacking, and according to a recent review the effects of this medication in rodent models of relapse to alcohol seeking are inconsistent^152^.

The effects of propranolol on disruption of memory reconsolidation seem to depend on activation of metabotropic glutamate class II receptor^153^. This is of interest, because both alcohol-dependent patients and rats show deficits in mGluR2 receptors within the mPFC^80^, which would predict increased stability of memories and in turn reduced cognitive flexibility. Rescue of this deficit through overexpression of mGluR2 in the IL was sufficient to restore control over alcohol-seeking behavior, and had no adverse effects in normal rats. Thus, alcohol-induced neurodegenerative processes in the mPFC that affect responsiveness of glutamatergic neurons are likely to interact with the dynamic formation of local ensembles that control alcohol-related behaviors in a stimulus-specific manner. Targeting metabotropic receptors such as mGluR2 with partial agonist or partial antagonist pharmacological tools may be a way to preferentially interact with aberrant hyperactive or hypoactive states of neurons while leaving the normal function largely unaffected.

The recent discovery that impaired GABA clearance in central amygdala is a causal factor behind pathological alcohol choice preference^128^ may also point to therapeutic strategies to rescue choice behavior, by recuing impaired GABA-clearance in the amygdala. Accomplishing this by directly targeting GAT-3 would require normalizing its expression or developing positive modulators of the transporter, drug actions for which there is currently no precedent. An approach that is more realistic in the short term is, however, suggested by recent observations. These have shown that presynaptic GABA-B receptors inhibit GABA release within the central amygdala^154^, and can therefore compensate for the excessive GABA-ergic inhibition that results in this brain area from impaired GABA-clearance. These findings, combined with our discovery that the impaired GABA-clearance promotes alcohol choice and compulsivity, point to a potential mechanism of action behind reports of beneficial effects obtained in alcoholism with the GABA-B agonist baclofen^35,39,155^.

Baclofen itself has inherent limitations as a therapeutic for alcohol addiction, and failed to obtain approval from the EMA for this indication. Because it is an orthosteric agonist, chronic administration of baclofen frequently results in tolerance to its effects, and a need for dose escalation^38,156^. This in turn results in a risk for lethal intoxication that has increased as the off-label use of baclofen for alcoholism has grown^37^. An attractive alternative approach is offered by positive allosteric modulators (PAMs) of the GABA-B receptor. This class of medications has the potential to achieve mechanistic and therapeutic effects similar to GABA-B agonists, while avoiding tolerance and overdose toxicity. We recently reported that the GABA-B PAM ADX71441 blocks stress-induced relapse to alcohol self-administration^157^. A key research question is whether the GABA-B PAM will also be able to normalize choice preference in the minority of rats that choose alcohol over a natural reward. Preliminary data suggests that this is indeed the case.

The therapeutic approaches discussed here should be taken as illustrative concepts, rather than specific clinical options, or drug development pathways. It is still early days. But some important takeaways to guide future treatment development efforts already seem to be emerging:

- The development of alcohol addiction certainly does not render patients completely insensitive to reward contingencies. It does, however, lead to the emergence of systematic biases in reward choice preferences. It is reasonable to assume that these reflect a persistent pathology of neurocircuitry that integrates outcome values and guides action selection. As a result, patients repeatedly make maladaptive choices, under conditions when people without an addiction decide advantageously. It is unclear to us why this pathophysiology would not qualify for being a disease mechanism.
- Alcohol use in patients with alcohol addiction represents a shift of choice behaviors away from non-drug rewards and toward alcohol. It seems unlikely to us that the brain pathology behind this shift can be understood through research strategies that study alcohol reinforcement in the absence of alternatives.
- The pathological shift in choice preferences, and continued use despite adverse consequences that are the hallmarks of alcohol addiction occurs only in a minority of alcohol users. It seems unlikely to us that it can be understood by studying behaviors that emerge in all animals tested.
- While worthwhile, identifying the vulnerable minority and using it for screening candidate therapeutics poses challenges when it come to throughput. A complementary approach in identifying novel therapeutic mechanisms may come from studies of the resilient majority.
- Using pharmacotherapeutic strategies that have activity-dependent effects in its infancy, but seems to offer a new area of exploration that may fruitfully combine behavioral and pharmacological interventions.
- Precision medicine is the road forward to improve outcomes in the treatment of alcohol addiction. Although tailoring medicines towards subpopulations presenting with distinct genetic factors and intermediate phenotypes seems counterintuitive to expensive medication development efforts, the issue at hand is to increase effect sizes towards levels that are clinically meaningful and thereby to gain patient and prescriber trust in such therapies. Their increased acceptance is likely to raise utilization and ultimately also sales.
